# Differences in neurophysiologic effects between CPAP and a novel high-flow therapy system

**DOI:** 10.1186/cc10740

**Published:** 2012-03-20

**Authors:** N Tiffin, S Connelly

**Affiliations:** 1 Special Care Technologies, Banbury, UK

## Introduction

CPAP therapy for respiratory insufficiency is an established and accepted mode of therapy; however, patient compliance remains an issue. Recent studies have shown that high-flow therapy (HFT), which uses high flows of warmed and humidified air/O_2 _mixtures through a nasal cannula, can also be effective in treating respiratory insufficiency. Although a nasal cannula is commonly preferred over a CPAP mask, patient comfort with HFT and CPAP has not been measured empirically. We sought to examine the autonomic neurophysiologic responses as a measure of comfort between these therapies.

## Methods

We used the Sensewear Armband (Bodymedia Inc., USA) to measure the Galvanic Skin Response (GSR) in 11 healthy volunteers (36 to 53 years). The 60-second averages of each test condition were made after 20 minutes of stabilization. Test conditions were pre and post baseline (no intervention), 10 cmH_2_O CPAP (Resmed, Sydney, Australia) and 15 LPM HFT (TNI, Würzburg, Germany) both in room air. Repeated ANOVA with *P *< 0.05.

## Results

**Figure 1:**
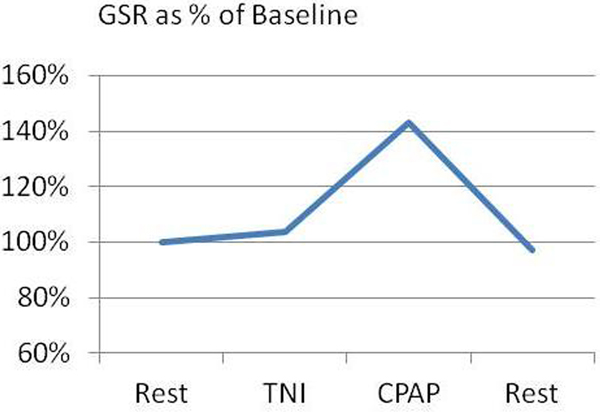


There were no statistically significant differences in GSR between pre and post baselines. CPAP produced an increase in GSR compared to both baselines (45%; *P *< 0.05) and to HFT (41%; *P *< 0.05). HFT produced no significant change in GSR compared to baseline. See Figure 1.

## Conclusion

GSR is a measurement of the sympathetic component of the autonomic nervous system. It is commonly referred to as the 'Fight or Flight' response, and when elevated indicates a state of psychological or physiological stress. Our data suggest that CPAP produces an increase in the GSR compared to rest, whilst TNI therapy produces no change in GSR compared to rest. This increased stress may lead to lower patient compliance when using CPAP therapy compared to TNI therapy, which has very high patient compliance rates.

